# Supplementary material for: STEA: Histologically Validated and Reference-Independent Major Cell-Type Annotation for Spatial Transcriptomics Reveals Relevant Cellular Organization and Architecture of Tumor Microenvironment
Source: Cancers (Basel). 2026 Apr 29;18(9):1425. doi: 10.3390/cancers18091425 (PMC13162884; doi:10.3390/cancers18091425)
Supplement: Supplementary file 1 [file cancers-18-01425-s001.zip › cancers-4235840-supplementary.pdf]

Supplementary Figures

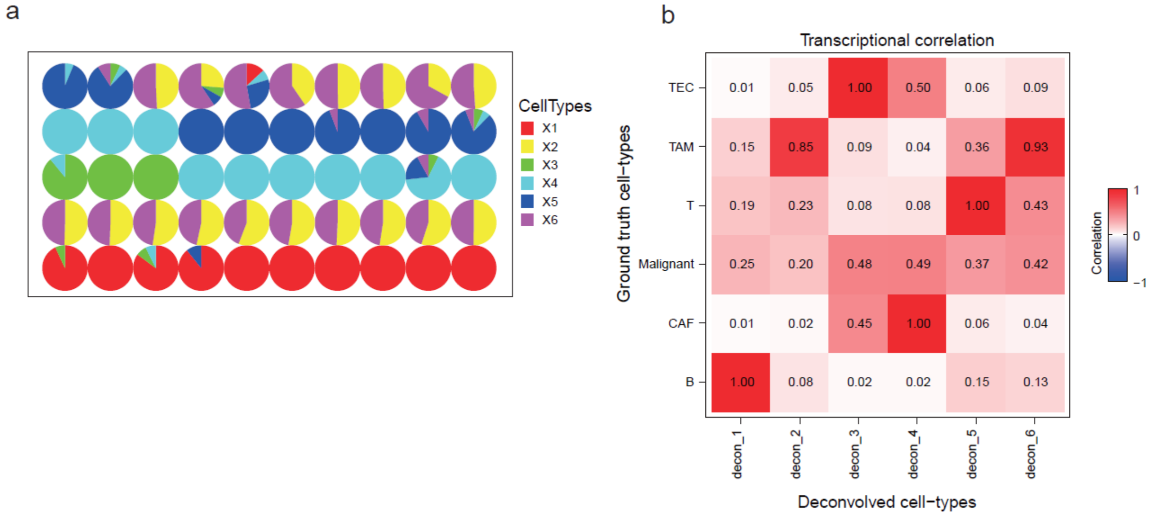

Figure S1. Deconvolution results of STdeconvolve on simulated sequencing-based HCC ST dataset. (a). The deconvolution results. (b). The heatmap illustrates the transcriptional correlation between ground truth cell types and deconvolved components.

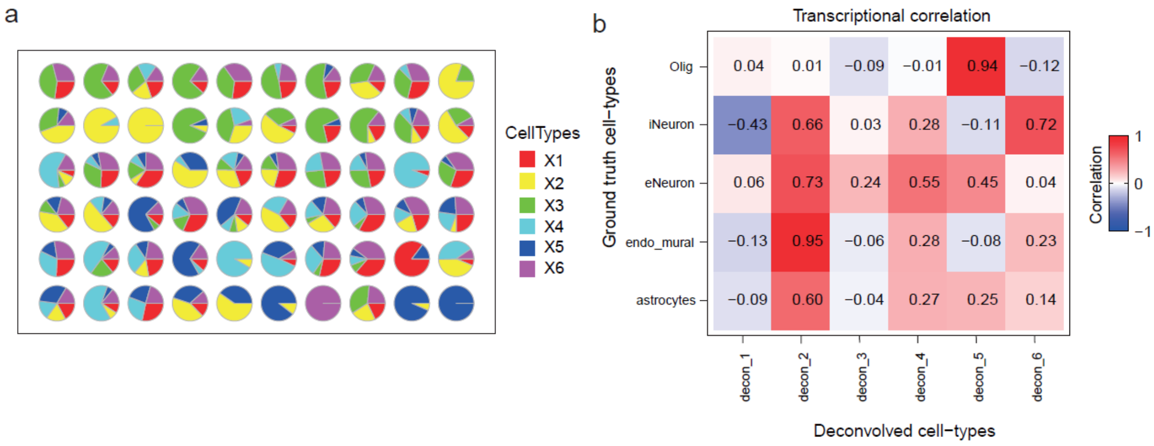

Figure S2. Deconvolution results of STdeconvolve on simulated imaged-based ST datasets. (a). The deconvolution results. (b). The heatmap illustrates the transcriptional correlation between ground truth cell types and deconvolved components.

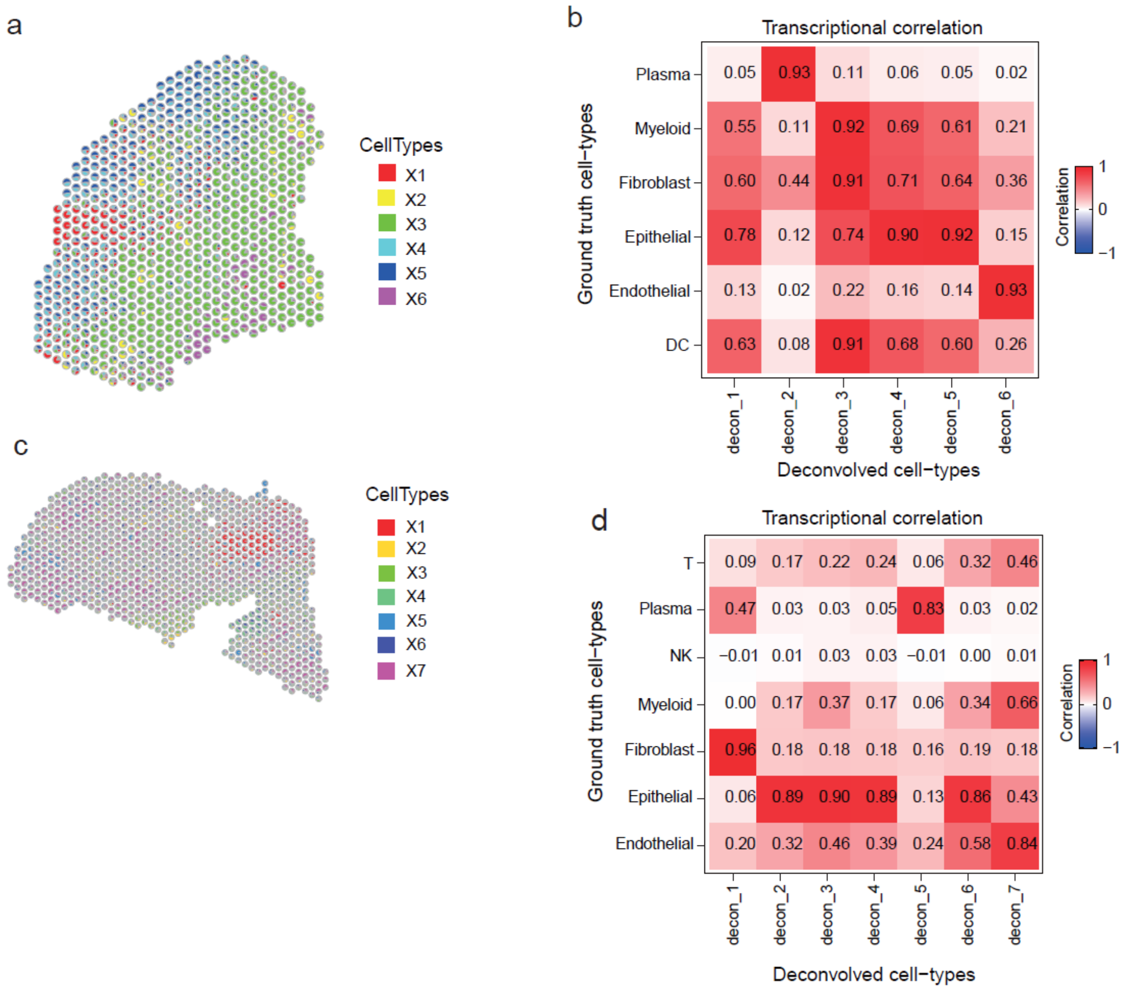

Figure S3. Deconvolution results of STdeconvolve on intraductal papillary mucinous neoplasm datasets. (a). The deconvolution results of sample 1. (b). The heatmap illustrates the transcriptional correlation between ground truth cell types and deconvolved components. (c). The deconvolution results of sample 2. (d). The heatmap illustrates the transcriptional correlation between ground truth cell types and deconvolved components.

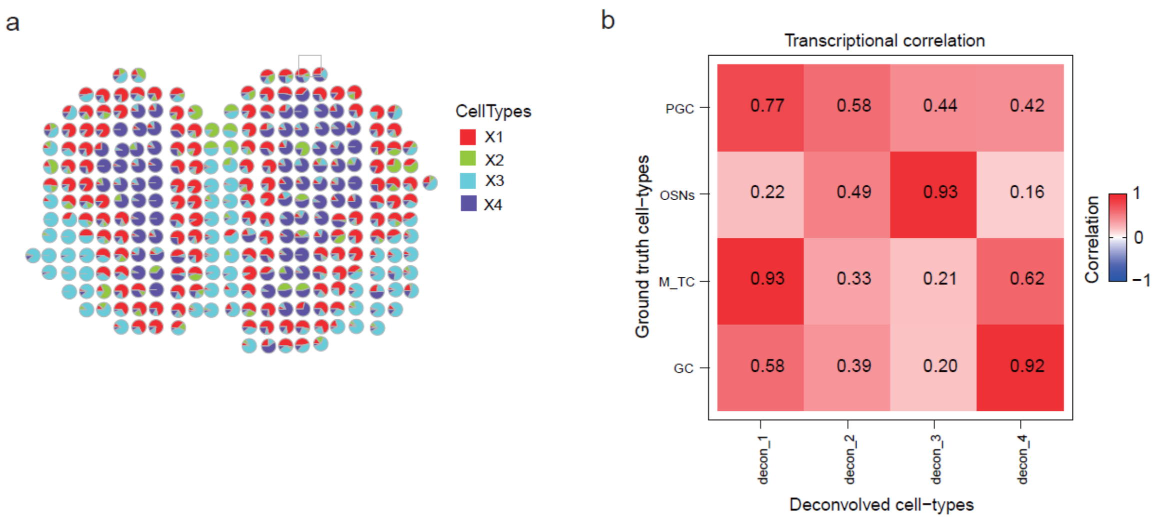

Figure S4. Deconvolution results of STdeconvolve on mouse olfactory bulb datasets. (a). The deconvolution results. (b). The heatmap illustrates the transcriptional correlation between ground truth cell types and deconvolved components.
